# Human Monocytic Ehrlichiosis, Mexico City, Mexico

**DOI:** 10.3201/eid2612.200520

**Published:** 2020-12

**Authors:** Virginia E. Alcántara-Rodríguez, Sokani Sánchez-Montes, Hugo Contreras, Pablo Colunga-Salas, Lauro Fierro-Flores, Sergio Avalos, Francisco Rodríguez-Rangel, Ingeborg Becker, David H. Walker

**Affiliations:** Secretaría de Salud de la Ciudad de México, Mexico City, Mexico (V.E. Alcántara-Rodríguez, H. Contreras, L. Fierro-Flores, S. Avalos, F. Rodríguez-Rangel);; Universidad Veracruzana, Veracruz, Mexico (S. Sánchez-Montes);; Universidad Nacional Autónoma de México, Mexico City (S. Sánchez-Montes, P. Colunga-Salas, I. Becker);; University of Texas Medical Branch, Galveston, Texas, USA (D.H. Walker)

**Keywords:** human monocytic ehrlichiosis, Ehrlichia chaffeensis, rickettsia, bacteria, man who was homeless, vector-borne infections, tick-borne infections, zoonoses, Mexico City, Mexico

## Abstract

Little information is available about human infections by the members of the genus *Ehrlichia* in Mexico. Only 2 species, *Ehrlichia canis* and *E. chaffensis*, are known to cause disease in this country. We report a fatal case of human monocytic ehrlichiosis in Mexico City in a man who was homeless.

The genus *Ehrlichia* contains 6 species of obligately intracytoplasmic bacteria that have major roles in human and veterinary medicine. These bacteria can cause ehrlichiosis, an emerging zoonoses transmitted mainly by bites of several hard tick species of the genera *Amblyomma*, *Ixodes*, and *Rhipicephalus* (*1*). In the Americas, the most relevant species that involves public health is *Ehrlichia chaffeensis*, the etiologic agent of human monocytic ehrlichiosis, an acute disease characterized by fever, thrombocytopenia, leukopenia, alterations of coagulation, and hepatic and neurologic involvement; systemic complications can lead to death in 3% of case-patients (*1**,**2*).

Little is known about *Ehrlichia* infections in Mexico. A human case was reported in the Yucatan Peninsula during 1999 in a male patient who had fever, anorexia, lymphadenopathy, cutaneous bleeding, and sore throat. Peripheral blood morulae, PCR detection of *E. chaffeensis*, and anemia were also reported (*3*). Subsequently, a case of *E. canis* infection was detected in the coastal state of Oaxaca in a veterinary stylist who had close contact with dogs (*4*). A study published in 2016 reported a female resident of the state of Mexico who had fever, thrombocytopenia, and alteration of liver enzyme levels and died after a long hospitalization (*5*). We report a fatal case of human monocytic ehrlichiosis in Mexico City, Mexico.

## The Study

On March 3, 2017, a 35-year-old man who was homeless (resident in Mexico City for 4 years) was admitted to the Emergency Department in the General Hospital of Xoco of the Ministry of Health of Mexico City because of trauma after a fall of 6 m from a bridge as a result of a suicide attempt. At admission, no lesions were detected in internal organs; a transtrochanteric fracture of the left femoral head was surgically repaired without complications. However, the patient had profuse bleeding during the surgical procedure, for which it was necessary to provide multiple blood transfusions. The blood units came from resident donors of Mexico City and the neighboring state of Mexico. In the postoperative period, the patient remained hospitalized for 65 days, during which behavioral alterations with several psychotic periods developed. He also had febrile episodes that evolved to a torpid state.

Routine laboratory analyses (blood count, blood chemistry, and serologic studies for infectious diseases) were performed. Because the patient was homeless and had persistent febrile episodes and dysfunction of the coagulation system, a possible infection by a rickettsial agent or *Bartonella* spp. was suspected because *Bartonella quintana* has been detected previously in human lice from persons who were homeless in Mexico City (*6*). For this reason 5 mL of whole blood was obtained, stored in EDTA, and processed for DNA extraction by using the QIAamp DNA Mini Kit (QIAGEN, https://www.qiagen.com) according to the manufacturer’s instructions. Blood was then examined for *Bartonella*, *Ehrlichia*/*Anaplasma*, and *Rickettsia* spp. by amplifying fragments of the *gltA, rrs*, and *sca5*, genes and using primers and PCR conditions specified (*7**–**9*). Positive controls (*E. canis* [GenBank accession no. MG917715], *B. vinsonii* [KT326174], and *Rickettsia amblyommatis* [KX363842) DNA]) were also included.

On April 27, 2017, antibodies against *Proteus* OX-19 at a titer of 1:320, leukocytosis, thrombocytosis, lymphopenia, anemia, hypoalbuminemia, and coagulation and liver enzyme alterations were reported (Table). The patient was given a diagnosis of septic shock and urosepsis and died on day 63 of hospitalization.

**Table:** Laboratory results for a patient with human monocytic ehrlichiosis, Mexico City, Mexico, 2017*

Day/date	Hematologic parameter												Blood chemistry parameter										Hepatic parameter										
	LEU, × 10^3^/μL	NEU, %	LYM, %	ERY, × 10^6^/μL	Hb, g/dL	HCT, %	MCV, fL	MCH, pg	ESR, mm/h	PLT, × 10^3^/μL	MPV, fL		GLU, mg/dL	Urea, mg/dL	CRE, mg/L	Na, mmol/L	K, mmol/L	Cl, mmol/L	Ca, mg/dL	Mg, mg/dL	IP, mg/dL		TB, mg/dL	DB, mg/dL	IB, mg/dL	PB, mg/dL	ALB, g/dL	AST, U/L	ALT, U/L	AP, U/L	GGT, U/L	GLB, g/dL	A/G
1/Mar 3	**18.5**	**91**	3.3	4.8	**14.4**	43.0	90	29	12.0	253	8.5		**139**	17	1.0	141	3.6	104	**8.9**	1.9	3.1		**2.2**	**0.4**	**1.8**	6	3.8	**51**	27	66	15	2.2	1.7
14/Mar 25	**20.0**	**92**	2.8	3.1	9.5	29.2	95	31	16.7	**616**	6.8		78	21	0.6	134	3.8	105	NR	NR	NR		**1.5**	**0.5**	**1.0**	4.8	2.7	**43**	**46**	76	**349**	2.1	1.3
16/Mar 27	10.7	78	**5.8**	2.4	7.5	22.0	92	30	16.5	**688**	6.3		76	41	0.8	140	4.1	**113**	**7.8**	1.7	4.0		**1.3**	**0.7**	0.6	4.3	2.2	**66**	38	75	35	2.1	1.0
18/Mar 29	**21.7**	**87**	3.3	3.2	9.6	29.4	92	33	18.1	**850**	6.8		72	32	0.8	141	4.6	**115**	**8.0**	1.8	3.8		**1.3**	**0.4**	**0.9**	4.1	1.8	**65**	38	80	35	2.3	0.8
20/Mar 31	**20.9**	**90**	3.2	3.4	**10.1**	31.0	90	29	17.9	**779**	7.3		99	21	0.6	141	3.5	**115**	**7.5**	1.6	2.7		NR	NR	NR	NR	NR	NR	NR	NR	NR	NR	NR
23/Apr 3	**12.5**	**86**	**5.5**	3.7	**10.6**	32.9	90	29	18.8	**523**	8.6		108	17	0.6	**146**	3.2	**113**	**7.2**	2.2	2.4		**1.1**	0.3	**0.8**	4	2.2	35	18	60	**68**	1.8	1.2
27/Apr 7	**16.4**	**83**	**9.4**	3.8	**11.3**	34.6	91	30	17.4	**688**	8.4		**185**	28	0.3	**146**	2.7	105	**7.5**	2.0	2.3		NR	NR	NR	NR	NR	NR	NR	NR	NR	NR	NR
37/Apr 17	11.2	69	**21.0**	3.5	**10.8**	32.0	90	34	18.0	**657**	7.7		**122**	26	0.4	135	4.0	104	**8.4**	1.8	3.8		0.5	0.2	0.3	5.8	2.4	**90**	**133**	**423**	**684**	3.4	0.7
48/Apr 28	**11.4**	76	**15.0**	2.6	7.7	23.0	89	33	17.4	**458**	7.8		NR	NR	NR	NR	NR	NR	NR	NR	NR		NR	NR	NR	NR	NR	NR	NR	NR	NR	NR	NR
52/ May 2	10.3	75	**14.0**	3.3	**9.9**	29.0	90	30	16.0	**710**	6.8		NR	NR	NR	NR	NR	NR	NR	NR	NR		NR	NR	NR	NR	NR	NR	NR	NR	NR	NR	NR
55/May 5	6.0	66	**22.0**	3.4	**9.9**	30.0	89	29	16.0	**606**	7.5		NR	NR	NR	NR	NR	NR	NR	NR	NR		NR	NR	NR	NR	NR	NR	NR	NR	NR	NR	NR
65/May 15	**32.7**	**94**	4.7	3.3	**9.6**	29.0	87	29	18.2	108	**12**		**112**	**66**	0.7	**150**	3.5	**120**	**7.4**	2.2	**4.9**		NR	NR	NR	NR	NR	NR	NR	NR	NR	NR	NR

*Bold indicates that the value is outside the reference range. A/G, albumin/globulin ratio; ALB, albumin; ALT, alanine aminotransferase; AP, alkaline phosphatase; AST, aspartate aminotransferase; Ca, calcium; Cl, chloride; CRE, creatinine; ERY, erythrocytes; DB, direct bilirubin; ESR, erythrocyte sedimentation rate; GGT, γ-glutamyltransferase; GLB, globulin; GLU, glucose; Hb, hemoglobin; HCT, hematocrit; IB, indirect bilirubin; IP, inorganic phosphate; K, potassium; LEU, leukocytes; LYM, lymphocytes; MCH, mean corpuscular hemoglobin; MCV, mean corpuscular volume; Mg, magnesium; MPV, mean platelet volume; Na, sodium; NEU, neutrophils; NR, no results; PB, partial bilirubin; PLT, platelets; TB, total bilirubin.

During hospitalization, leukocytosis developed, which might have been associated with the multiple traumas. The patient initially had a platelet count within the reference range, but thrombocytosis developed, and the platelet count increased to 850,000/μL. However, with persistent leukocytosis, the platelet count dropped, and thrombocytopenia (108,000/μL) developed shortly before death.

Total serum protein and albumin concentrations at admission were within reference ranges, but during hospitalization they decreased, as would be expected for a diagnosis of ehrlichiosis. Alterations in liver enzyme levels and coagulation times also developed. Levels of alanine aminotransferase (ALT) and aspartate aminotransferase also increased after admission; ALT showed the largest increase. The level of γ-glutamyl transpeptidase increased by >10 times over its reference value (Table). The increase in the level of ALT could be related to hepatic alterations linked to ehrlichiosis.

Necropsy showed hepatosplenomegaly and pleural effusions. Molecular assays did not detect *Bartonella* or *Rickettsia* spp., but they did detect *Ehrlichia/Anaplasma* spp. when primers Ehr1/Ehr2 were used. We isolated a 400-bp fragment of the 16S rRNA gene, which showed 99% identity with that of the *E. chaffensis* strain Arkansas. In addition, a 500-bp fragment of the *dsb* gene (present only in the members of the genus *Ehrlichia*) was amplified (*10*) and showed 100% identity with the sequence of *E. chaffensis* strain Arkansas.

Phylogenetic analysis of the isolated sequences grouped them with sequences of 2 strains of *E. chaffensis* (strain Arkansas and West Paces) detected in the United States; these sequences had a bootstrap value of 99. The sequences we obtained were deposited in GenBank under accession numbers MK351589 and MK370999 (Figure).

**Figure:**
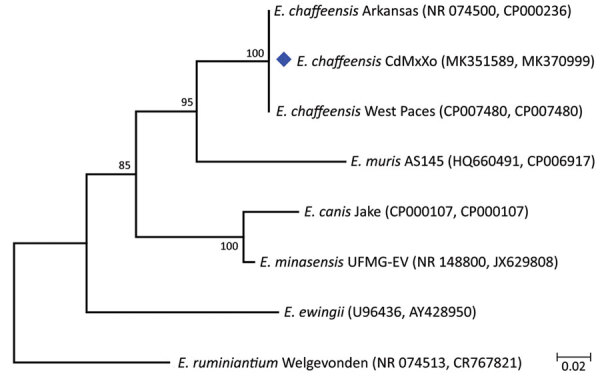
Maximum-likelihood phylogenetic tree for *Ehrlichia chaffensis* from a patient with human monocytic ehrlichiosis, Mexico City, Mexico (blue diamond), and reference sequences. The tree was generated by using concatenated fragments of 16S rRNA and *dsb* genes in an 850-bp alignment. GenBank accession numbers are indicated in parentheses. Numbers along branches are bootstrap values. Scale bar indicates nucleotide substitutions per site.

## Conclusions

Ehrlichioses represent systemic infections that can cause damage to different organs and systems, affecting the liver, meninges, brain, heart, and lungs. Because the pathophysiology of this disease is not well established, the findings obtained by necropsy are useful. During his hospitalization, the patient acquired some bacterial infections, which could explain the paradox that he initially had thrombocytosis and leukocytosis instead of thrombocytopenia and leukopenia, as would be expected for ehrlichiosis. Leukopenia and thrombocytopenia are present in >50% of patients given a diagnosis of *E. chaffeensis* infection (*5*).

The patient might have been infected by blood transfusion because the long period between hospital admission and detection of thrombocytopenia is compatible with the incubation period for infection with *E. chaffeensis*. The patient received multiple blood transfusions from donors in Mexico City and the state of Mexico; in this state, a fatal case of ehrlichiosis caused by blood transfusion has been reported (*5*). A study in Costa Rica reported a large number of *E. canis*–infected healthy blood donors (*11*), and a study in the United States reported a patient who was infected by blood transfusion containing *E. ewingii* (*12*). More recently, a potential case of transfusion-transmitted human monocytic ehrlichiosis was reported in a 59-year-old woman in the United States after she received a blood stem cell transplant (*13*).

Although this possibility is less likely, it cannot be ruled out that ehrlichiosis could be acquired by tick bite. *E. chaffeensis* was previously detected in 3 hard tick species (*Amblyomma americanum*, *A. mixtum*, and *Rhipicephalus sanguineus* sensu 1ato) (*14*) in Mexico. Only the lone star tick (*A. americanum*) has been implicated as a primary vector of this pathogen in the United States; however, its presence in Mexico has only been recorded restricted to the Nearctic region (*15*). Conversely, no studies demonstrate the role of the other 2 tick species as potential vectors of this pathogen or whether DNA of this pathogen came from an infected host from which these ticks fed.

For this reason, it is essential to perform systematic surveillance of this and other tick-borne pathogens in blood donors in Mexico. Studies should also be conducted with questing ticks to identify the risk to which the population is exposed and establish the actual distribution of the pathogen.
